# 3,4′,5-Trichloro­biphenyl-4-yl 2,2,2-trichloro­ethyl sulfate

**DOI:** 10.1107/S1600536813007976

**Published:** 2013-03-28

**Authors:** Hans-Joachim Lehmler, Xianran He, Michael W. Duffel, Sean Parkin

**Affiliations:** a The University of Iowa, Department of Occupational and Environmental Health, Iowa City, IA 52242, USA; b The University of Iowa, Department of Pharmaceutical Sciences and Experimental Therapeutics, Iowa City, IA 52242, USA; c University of Kentucky, Department of Chemistry, Lexington, KY 40506-0055, USA

## Abstract

Crystals of the title compound, C_14_H_8_Cl_6_O_4_S, are twinned by inversion, with unequal components [0.85 (3):0.15 (3)]. The asymmetric unit contains two independent mol­ecules that are related by a pseudo-inversion center. The C_ar_—O [1.393 (9) and 1.397 (9) Å] and ester S—O bond lengths [1.600 (5) and 1.590 (5) Å] of both mol­ecules are comparable to the structurally related 2,3,5,5-trichloro­biphenyl-4-yl 2,2,2-trichloro­ethyl sulfate. The dihedral angles between the benzene rings in the two mol­ecules are 37.8 (2) and 35.0 (2)°.

## Related literature

For related structures of biphenyl-4-yl ester 2,2,2-trichloro-ethyl esters of sulfuric acid, see: Li *et al.* (2008 ▶, 2010*a* ▶,*b* ▶,*c* ▶). For a review of structures of sulfuric acid aryl mono esters, see: Brandao *et al.* (2005 ▶); Denehy *et al.* (2006 ▶). For additional background to sulfate metabolites of polychlorinated bi­phenyls, see: Liu *et al.* (2006 ▶, 2009 ▶); Wang *et al.* (2006 ▶); Dhakal *et al.* (2012 ▶); Zhai *et al.* (2013 ▶).
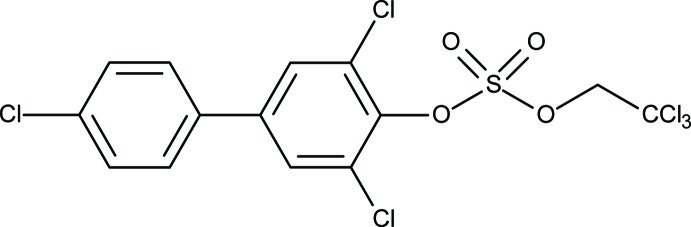


## Experimental

### 

#### Crystal data

C_14_H_8_Cl_6_O_4_S
*M* *_r_* = 484.96
Orthorhombic, 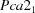

*a* = 13.993 (3) Å
*b* = 9.1890 (18) Å
*c* = 28.778 (6) Å
*V* = 3700.3 (13) Å^3^
*Z* = 8
Cu *K*α radiation
μ = 9.71 mm^−1^
*T* = 90 K
0.17 × 0.09 × 0.02 mm

#### Data collection

Bruker X8 Proteum diffractometer
Absorption correction: multi-scan (*SADABS*; Bruker, 2006 ▶) *T*_min_ = 0.504, *T*_max_ = 0.830
45894 measured reflections
6651 independent reflections
6238 reflections with *I* > 2σ(*I*)
*R*_int_ = 0.062

#### Refinement

*R*[*F* ^2^ > 2σ(*F* ^2^)] = 0.064
*wR*(*F* ^2^) = 0.161
*S* = 1.15
6651 reflections
302 parameters
1 restraint
H-atom parameters constrained
Δρ_max_ = 0.96 e Å^−3^
Δρ_min_ = −0.85 e Å^−3^
Absolute structure: Flack (1983 ▶), 3176 Friedel pairs
Flack parameter: 0.15 (3)

### 

Data collection: *APEX2* (Bruker, 2006 ▶); cell refinement: *SAINT* (Bruker, 2006 ▶); data reduction: *SAINT*; program(s) used to solve structure: *SHELXS97* (Sheldrick, 2008 ▶); program(s) used to refine structure: *SHELXL97* (Sheldrick, 2008 ▶); molecular graphics: *XP* in *SHELXTL* (Sheldrick, 2008 ▶); software used to prepare material for publication: *SHELXL97* and local procedures.

## Supplementary Material

Crystal structure: contains datablock(s) I, global. DOI: 10.1107/S1600536813007976/yk2088sup1.cif

Structure factors: contains datablock(s) I. DOI: 10.1107/S1600536813007976/yk2088Isup2.hkl

Supplementary material file. DOI: 10.1107/S1600536813007976/yk2088Isup3.cml

Additional supplementary materials:  crystallographic information; 3D view; checkCIF report

## Figures and Tables

**Fig. 1.**
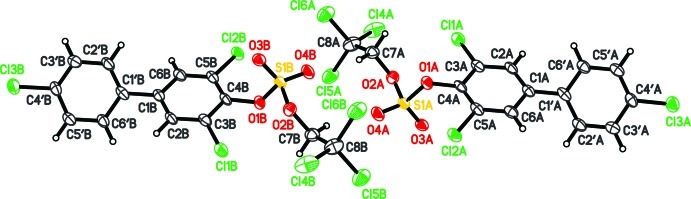
View of the title compound showing the atom-labeling scheme. Displacement ellipsoids are drawn at the 50% probability level.

## supplementary crystallographic information

### Comment

Sulfuric acid monoesters of hydroxylated polychlorinated biphenyls (OHPCBs) are emerging as an important class of metabolites of polychlorinated biphenyls (PCBs). Two recent *in vivo* studies report the formation of PCB sulfates by rats (Dhakal *et al.*, 2012) and poplar plants (Zhai *et al.*, 2013). *In vitro* studies demonstrate that PCB sulfates are both substrates and inhibitors of mammalian cytosolic sulfotransferases (Liu *et al.*, 2006; Wang *et al.*, 2006; Liu *et al.*, 2009). Only limited structural information about sulfate mono- and diesters of hydroxylated PCBs is available to support structure-activity or structure-property relationship studies. Here we report the structure of the title compound, a biphenyl-4-yl 2,2,2-trichloroethyl sulfate with two chlorine substituents *ortho* to the sulfate group, to contribute to the number of available crystal structures.

The two independent molecules of the title compound in the asymmetric unit are related by a pseudo-inversion center. The length of the C_aromatic_—O bonds of the two molecules are 1.393 (9) and 1.397 (9) Å, respectively. These bond lengths are comparable to the C_aromatic_—O bond length (1.405 Å) reported for the structurally related 2',3,5,5'-trichloro-biphenyl-4-yl 2,2,2-trichloroethyl sulfate (Li *et al.*, 2010*b*). In contrast, biphenyl-4-yl 2,2,2-trichloroethyl sulfates without electronegative chlorine substituents *ortho* to the sulfate group have slightly longer C_aromatic_—O bond length ranging from 1.426 to 1.449 Å (Li *et al.*, 2008; Li *et al.*, 2010*b*; Li *et al.*, 2010*a*; Li *et al.*, 2010*c*).

The lengths of the PCB sulfate ester bond of the title compound (*i.e.*, S1—O1) are 1.600 (5) and 1.590 (5) Å. In contrast, biphenyl-4-yl 2,2,2-trichloroethyl sulfates without chlorine substituents *ortho* to the sulfate group typically have shorter sulfate ester bond lengths ranging from 1.563 to 1.586 Å (Li *et al.*, 2008; Li *et al.*, 2010*b*; Li *et al.*, 2010*a*; Li *et al.*, 2010*c*). Similar to aromatic sulfate monoesters (Brandao *et al.*, 2005; Denehy *et al.*, 2006), this difference suggests that chlorine substituents *ortho* to the sulfate group decrease the stability of the S—O ester bond.

The dihedral angle of the biphenyl moiety of PCB derivatives is a structural parameter associated with the affinity of PCB derivatives for cellular target molecules. The two molecules of the title compound have solid state dihedral angles of 37.8 (2)° and 35.0 (2)°. Similarly, structurally related biphenyl-4-yl 2,2,2-trichloroethyl sulfates have dihedral angles ranging from 4.9° to 41.8° in the solid state (Li *et al.*, 2008; Li *et al.*, 2010*a*; Li *et al.*, 2010*c*). The fact that biphenyl-4-yl 2,2,2-trichloroethyl sulfates without *ortho* chlorine substituents adopt a range of dihedral angles can be explained by crystal packing effects, which force the biphenyl moiety to adopt an energetically less favorable conformation in the solid state.

### Experimental

The title compound was synthesized from 3,4',5-trichlorobiphenyl-4-ol and 2,2,2-trichloroethyl sulfonyl chloride using 4-dimethylaminopyridine as catalyst as reported previously (Li *et al.*, 2008). Crystals suitable for X-ray diffraction analysis were obtained by slow evaporation of a methanolic solution.

### Refinement

H atoms were found in difference Fourier maps and subsequently placed in idealized positions with constrained distances of 0.99 Å (*R*_2_CH_2_), 0.95 Å (C_sp2_H), and with *U*_iso_(H) values set to either 1.2*U*_eq_ or 1.5*U*_eq_ (RCH_3_, OH) of the attached atom.

The two independent molecules are related by a pseudo-inversion centre, which results in large correlations between the displacement parameters. In order to ensure satisfactory refinement, the displacement parameters of equivalent atoms in each molecule were constrained to be the same using the EADP command of *SHELXL97*.

### Crystal data

**Table:**

C_14_H_8_Cl_6_O_4_S	*F*(000) = 1936
*M**_r_* = 484.96	*D*_x_ = 1.741 Mg m^−^^3^
Orthorhombic, *P**c**a*2_1_	Cu *K*α radiation, λ = 1.54178 Å
Hall symbol: P 2c -2ac	Cell parameters from 9992 reflections
*a* = 13.993 (3) Å	θ = 3.1–68.3°
*b* = 9.1890 (18) Å	µ = 9.71 mm^−^^1^
*c* = 28.778 (6) Å	*T* = 90 K
*V* = 3700.3 (13) Å^3^	Flake, colourless
*Z* = 8	0.17 × 0.09 × 0.02 mm

### Data collection

**Table:**

Bruker X8 Proteum diffractometer	6651 independent reflections
Radiation source: fine-focus rotating anode	6238 reflections with *I* > 2σ(*I*)
Graded multilayer optics monochromator	*R*_int_ = 0.062
Detector resolution: 5.6 pixels mm^-1^	θ_max_ = 68.4°, θ_min_ = 3.1°
φ and ω scans	*h* = −14→16
Absorption correction: multi-scan (*SADABS*; Bruker, 2006)	*k* = −10→11
*T*_min_ = 0.504, *T*_max_ = 0.830	*l* = −34→34
45894 measured reflections	

### Refinement

**Table:**

Refinement on *F*^2^	Secondary atom site location: difference Fourier map
Least-squares matrix: full	Hydrogen site location: inferred from neighbouring sites
*R*[*F*^2^ > 2σ(*F*^2^)] = 0.064	H-atom parameters constrained
*wR*(*F*^2^) = 0.161	*w* = 1/[σ^2^(*F*_o_^2^) + (0.0514*P*)^2^ + 21.3733*P*] where *P* = (*F*_o_^2^ + 2*F*_c_^2^)/3
*S* = 1.15	(Δ/σ)_max_ < 0.001
6651 reflections	Δρ_max_ = 0.96 e Å^−^^3^
302 parameters	Δρ_min_ = −0.85 e Å^−^^3^
1 restraint	Absolute structure: Flack (1983), 3176 Friedel pairs
Primary atom site location: structure-invariant direct methods	Flack parameter: 0.15 (3)

### Special details

**Table:**

Experimental. The crystal was twinned by inversion, but with unequal sized pieces of each component. The refined Flack parameter indicates major:minor fractions of 0.85 (3):0.15 (3).
Geometry. All s.u.'s (except the s.u. in the dihedral angle between two l.s. planes) are estimated using the full covariance matrix. The cell s.u.'s are taken into account individually in the estimation of s.u.'s in distances, angles and torsion angles; correlations between s.u.'s in cell parameters are only used when they are defined by crystal symmetry. An approximate (isotropic) treatment of cell s.u.'s is used for estimating s.u.'s involving l.s. planes.
Refinement. Refinement of *F*^2^ against all reflections. The weighted *R*-value *wR* and goodness of fit *S* are based on *F*^2^. Conventional *R*-values *R* are based on *F*, with *F* set to zero for negative *F*^2^. The threshold expression of *F*^2^ > 2σ(*F*^2^) is used only for calculating *R*-factors(gt) *etc*. and is not relevant to the choice of reflections for refinement. *R*-values based on *F*^2^ are statistically about twice as large as those based on *F*, and *R*-values based on ALL data will be even larger.

### Fractional atomic coordinates and isotropic or equivalent isotropic displacement parameters (Å^2^)

**Table:**

	*x*	*y*	*z*	*U*_iso_*/*U*_eq_	
S1A	0.77740 (12)	0.4047 (2)	0.35290 (7)	0.0283 (2)	
O1A	0.8439 (4)	0.2785 (5)	0.33235 (19)	0.0273 (7)	
O2A	0.8158 (4)	0.4167 (6)	0.40377 (19)	0.0313 (7)	
O3A	0.7985 (4)	0.5393 (6)	0.3314 (2)	0.0302 (7)	
O4A	0.6838 (4)	0.3483 (6)	0.3531 (2)	0.0349 (7)	
Cl1A	1.03327 (13)	0.3420 (2)	0.37524 (7)	0.0380 (3)	
Cl2A	0.78298 (12)	0.2737 (2)	0.23480 (7)	0.0318 (2)	
Cl3A	1.37897 (13)	0.5084 (2)	0.10448 (8)	0.0378 (3)	
Cl4A	0.8960 (2)	0.4431 (3)	0.50012 (9)	0.0563 (4)	
Cl5A	0.69326 (19)	0.4810 (2)	0.48583 (9)	0.0487 (4)	
Cl6A	0.76595 (19)	0.2066 (2)	0.52014 (8)	0.0489 (4)	
C1A	1.0641 (5)	0.3630 (7)	0.2372 (3)	0.0240 (9)	
C2A	1.0822 (5)	0.3612 (8)	0.2847 (3)	0.0273 (9)	
H2A	1.1456	0.3760	0.2956	0.033*	
C3A	1.0090 (5)	0.3381 (8)	0.3166 (3)	0.0276 (9)	
C4A	0.9162 (5)	0.3143 (8)	0.3011 (3)	0.0252 (9)	
C5A	0.8987 (5)	0.3119 (7)	0.2542 (3)	0.0259 (9)	
C6A	0.9704 (5)	0.3381 (8)	0.2223 (3)	0.0273 (9)	
H6A	0.9560	0.3393	0.1900	0.033*	
C7A	0.7997 (6)	0.2919 (9)	0.4344 (3)	0.0326 (10)	
H7A1	0.8546	0.2241	0.4332	0.039*	
H7A2	0.7412	0.2386	0.4251	0.039*	
C8A	0.7887 (7)	0.3548 (10)	0.4822 (3)	0.0431 (12)	
C1'A	1.1400 (5)	0.3932 (8)	0.2034 (3)	0.0284 (9)	
C2'A	1.1233 (5)	0.4783 (9)	0.1639 (3)	0.0314 (11)	
H2'A	1.0607	0.5138	0.1581	0.038*	
C3'A	1.1959 (5)	0.5118 (9)	0.1333 (3)	0.0307 (10)	
H3'A	1.1833	0.5691	0.1065	0.037*	
C4'A	1.2877 (5)	0.4609 (9)	0.1420 (3)	0.0284 (10)	
C5'A	1.3053 (5)	0.3752 (8)	0.1800 (3)	0.0293 (10)	
H5'A	1.3677	0.3379	0.1850	0.035*	
C6'A	1.2329 (5)	0.3424 (8)	0.2113 (3)	0.0285 (9)	
H6'A	1.2464	0.2854	0.2380	0.034*	
S1B	0.48992 (12)	0.0952 (2)	0.44025 (7)	0.0283 (2)	
O1B	0.4243 (3)	0.2207 (5)	0.46086 (19)	0.0273 (7)	
O2B	0.4512 (4)	0.0835 (6)	0.38944 (19)	0.0313 (7)	
O3B	0.4667 (4)	−0.0367 (6)	0.4615 (2)	0.0302 (7)	
O4B	0.5834 (4)	0.1524 (6)	0.4402 (2)	0.0349 (7)	
Cl1B	0.23508 (13)	0.1574 (2)	0.41731 (7)	0.0380 (3)	
Cl2B	0.48229 (12)	0.2267 (2)	0.55864 (7)	0.0318 (2)	
Cl3B	−0.11866 (13)	0.0187 (2)	0.68821 (8)	0.0378 (3)	
Cl4B	0.3715 (2)	0.0537 (3)	0.29351 (9)	0.0563 (4)	
Cl5B	0.50048 (19)	0.2929 (2)	0.27257 (8)	0.0487 (4)	
Cl6B	0.57633 (19)	0.0222 (3)	0.30941 (8)	0.0489 (4)	
C1B	0.2034 (5)	0.1376 (8)	0.5547 (3)	0.0240 (9)	
C2B	0.1855 (5)	0.1373 (8)	0.5077 (3)	0.0273 (9)	
H2B	0.1222	0.1221	0.4967	0.033*	
C3B	0.2590 (5)	0.1590 (8)	0.4759 (3)	0.0276 (9)	
C4B	0.3515 (5)	0.1866 (8)	0.4923 (3)	0.0252 (9)	
C5B	0.3684 (5)	0.1883 (8)	0.5392 (3)	0.0259 (9)	
C6B	0.2963 (5)	0.1672 (8)	0.5714 (3)	0.0273 (9)	
H6B	0.3089	0.1725	0.6038	0.033*	
C7B	0.4686 (6)	0.2041 (9)	0.3589 (3)	0.0326 (10)	
H7B1	0.5276	0.2556	0.3684	0.039*	
H7B2	0.4146	0.2736	0.3604	0.039*	
C8B	0.4791 (7)	0.1461 (10)	0.3102 (3)	0.0431 (12)	
C1'B	0.1230 (5)	0.1095 (8)	0.5886 (3)	0.0284 (9)	
C2'B	0.1395 (5)	0.0364 (9)	0.6294 (3)	0.0314 (11)	
H2'B	0.2024	0.0042	0.6364	0.038*	
C3'B	0.0659 (5)	0.0087 (8)	0.6607 (3)	0.0307 (10)	
H3'B	0.0784	−0.0421	0.6888	0.037*	
C4'B	−0.0243 (5)	0.0551 (9)	0.6506 (3)	0.0284 (10)	
C5'B	−0.0442 (5)	0.1307 (8)	0.6098 (3)	0.0293 (10)	
H5'B	−0.1072	0.1629	0.6031	0.035*	
C6'B	0.0308 (5)	0.1580 (8)	0.5789 (3)	0.0285 (9)	
H6'B	0.0188	0.2102	0.5510	0.034*	

### Atomic displacement parameters (Å^2^)

**Table:**

	*U*^11^	*U*^22^	*U*^33^	*U*^12^	*U*^13^	*U*^23^
S1A	0.0183 (5)	0.0276 (5)	0.0392 (6)	0.0017 (4)	0.0002 (4)	0.0039 (4)
O1A	0.0202 (14)	0.0205 (15)	0.0411 (17)	−0.0046 (12)	0.0037 (13)	0.0031 (12)
O2A	0.0285 (16)	0.0267 (16)	0.0386 (17)	−0.0036 (13)	0.0023 (13)	0.0059 (13)
O3A	0.0248 (17)	0.0217 (16)	0.0442 (18)	0.0022 (12)	0.0006 (13)	0.0063 (13)
O4A	0.0187 (15)	0.0370 (18)	0.0491 (19)	−0.0033 (13)	−0.0001 (14)	0.0041 (15)
Cl1A	0.0219 (5)	0.0543 (7)	0.0379 (6)	−0.0016 (5)	−0.0044 (5)	0.0037 (5)
Cl2A	0.0167 (5)	0.0340 (6)	0.0447 (6)	−0.0060 (4)	−0.0047 (4)	0.0021 (5)
Cl3A	0.0279 (6)	0.0455 (7)	0.0399 (6)	−0.0032 (5)	0.0037 (5)	0.0000 (6)
Cl4A	0.0802 (11)	0.0341 (6)	0.0545 (8)	−0.0139 (7)	−0.0252 (7)	0.0035 (5)
Cl5A	0.0709 (11)	0.0286 (7)	0.0465 (9)	0.0093 (7)	0.0109 (8)	0.0067 (7)
Cl6A	0.0715 (11)	0.0299 (8)	0.0454 (9)	0.0031 (7)	0.0054 (8)	0.0030 (7)
C1A	0.018 (2)	0.0134 (18)	0.040 (2)	0.0007 (16)	0.0015 (18)	0.0006 (17)
C2A	0.0137 (19)	0.023 (2)	0.045 (3)	−0.0001 (16)	−0.0014 (17)	0.0013 (19)
C3A	0.019 (2)	0.020 (2)	0.044 (2)	0.0014 (17)	−0.0017 (18)	0.0002 (18)
C4A	0.0122 (18)	0.0156 (19)	0.048 (3)	−0.0009 (15)	−0.0002 (18)	0.0014 (17)
C5A	0.017 (2)	0.0120 (18)	0.048 (3)	0.0000 (16)	0.0010 (18)	0.0004 (17)
C6A	0.020 (2)	0.022 (2)	0.040 (2)	−0.0008 (17)	−0.0039 (18)	−0.0016 (18)
C7A	0.033 (2)	0.027 (2)	0.038 (2)	0.001 (2)	−0.002 (2)	0.0039 (19)
C8A	0.060 (3)	0.028 (2)	0.041 (3)	−0.002 (2)	−0.007 (3)	0.000 (2)
C1'A	0.017 (2)	0.024 (2)	0.044 (2)	0.0023 (17)	−0.0018 (18)	−0.0069 (19)
C2'A	0.017 (2)	0.034 (3)	0.043 (2)	0.0000 (19)	−0.0038 (19)	0.000 (2)
C3'A	0.025 (2)	0.030 (2)	0.037 (2)	0.002 (2)	−0.005 (2)	0.000 (2)
C4'A	0.019 (2)	0.026 (2)	0.040 (2)	−0.0023 (18)	0.0028 (18)	−0.0037 (19)
C5'A	0.019 (2)	0.026 (2)	0.043 (3)	0.0008 (18)	−0.0034 (18)	−0.003 (2)
C6'A	0.019 (2)	0.023 (2)	0.042 (3)	−0.0013 (17)	−0.0001 (19)	0.001 (2)
S1B	0.0183 (5)	0.0276 (5)	0.0392 (6)	0.0017 (4)	0.0002 (4)	0.0039 (4)
O1B	0.0202 (14)	0.0205 (15)	0.0411 (17)	−0.0046 (12)	0.0037 (13)	0.0031 (12)
O2B	0.0285 (16)	0.0267 (16)	0.0386 (17)	−0.0036 (13)	0.0023 (13)	0.0059 (13)
O3B	0.0248 (17)	0.0217 (16)	0.0442 (18)	0.0022 (12)	0.0006 (13)	0.0063 (13)
O4B	0.0187 (15)	0.0370 (18)	0.0491 (19)	−0.0033 (13)	−0.0001 (14)	0.0041 (15)
Cl1B	0.0219 (5)	0.0543 (7)	0.0379 (6)	−0.0016 (5)	−0.0044 (5)	0.0037 (5)
Cl2B	0.0167 (5)	0.0340 (6)	0.0447 (6)	−0.0060 (4)	−0.0047 (4)	0.0021 (5)
Cl3B	0.0279 (6)	0.0455 (7)	0.0399 (6)	−0.0032 (5)	0.0037 (5)	0.0000 (6)
Cl4B	0.0802 (11)	0.0341 (6)	0.0545 (8)	−0.0139 (7)	−0.0252 (7)	0.0035 (5)
Cl5B	0.0709 (11)	0.0286 (7)	0.0465 (9)	0.0093 (7)	0.0109 (8)	0.0067 (7)
Cl6B	0.0715 (11)	0.0299 (8)	0.0454 (9)	0.0031 (7)	0.0054 (8)	0.0030 (7)
C1B	0.018 (2)	0.0134 (18)	0.040 (2)	0.0007 (16)	0.0015 (18)	0.0006 (17)
C2B	0.0137 (19)	0.023 (2)	0.045 (3)	−0.0001 (16)	−0.0014 (17)	0.0013 (19)
C3B	0.019 (2)	0.020 (2)	0.044 (2)	0.0014 (17)	−0.0017 (18)	0.0002 (18)
C4B	0.0122 (18)	0.0156 (19)	0.048 (3)	−0.0009 (15)	−0.0002 (18)	0.0014 (17)
C5B	0.017 (2)	0.0120 (18)	0.048 (3)	0.0000 (16)	0.0010 (18)	0.0004 (17)
C6B	0.020 (2)	0.022 (2)	0.040 (2)	−0.0008 (17)	−0.0039 (18)	−0.0016 (18)
C7B	0.033 (2)	0.027 (2)	0.038 (2)	0.001 (2)	−0.002 (2)	0.0039 (19)
C8B	0.060 (3)	0.028 (2)	0.041 (3)	−0.002 (2)	−0.007 (3)	0.000 (2)
C1'B	0.017 (2)	0.024 (2)	0.044 (2)	0.0023 (17)	−0.0018 (18)	−0.0069 (19)
C2'B	0.017 (2)	0.034 (3)	0.043 (2)	0.0000 (19)	−0.0038 (19)	0.000 (2)
C3'B	0.025 (2)	0.030 (2)	0.037 (2)	0.002 (2)	−0.005 (2)	0.000 (2)
C4'B	0.019 (2)	0.026 (2)	0.040 (2)	−0.0023 (18)	0.0028 (18)	−0.0037 (19)
C5'B	0.019 (2)	0.026 (2)	0.043 (3)	0.0008 (18)	−0.0034 (18)	−0.003 (2)
C6'B	0.019 (2)	0.023 (2)	0.042 (3)	−0.0013 (17)	−0.0001 (19)	0.001 (2)

### Geometric parameters (Å, º)

**Table:**

S1A—O4A	1.408 (5)	S1B—O3B	1.396 (6)
S1A—O3A	1.414 (6)	S1B—O4B	1.409 (5)
S1A—O2A	1.563 (6)	S1B—O2B	1.563 (6)
S1A—O1A	1.600 (5)	S1B—O1B	1.590 (5)
O1A—C4A	1.393 (9)	O1B—C4B	1.397 (9)
O2A—C7A	1.464 (9)	O2B—C7B	1.434 (9)
Cl1A—C3A	1.722 (9)	Cl1B—C3B	1.720 (8)
Cl2A—C5A	1.749 (7)	Cl2B—C5B	1.726 (7)
Cl3A—C4'A	1.730 (8)	Cl3B—C4'B	1.739 (8)
Cl4A—C8A	1.783 (10)	Cl4B—C8B	1.793 (10)
Cl5A—C8A	1.772 (10)	Cl5B—C8B	1.755 (9)
Cl6A—C8A	1.774 (9)	Cl6B—C8B	1.774 (10)
C1A—C2A	1.391 (11)	C1B—C2B	1.378 (11)
C1A—C6A	1.400 (10)	C1B—C6B	1.412 (10)
C1A—C1'A	1.467 (10)	C1B—C1'B	1.510 (10)
C2A—C3A	1.390 (11)	C2B—C3B	1.389 (11)
C2A—H2A	0.9500	C2B—H2B	0.9500
C3A—C4A	1.390 (10)	C3B—C4B	1.401 (10)
C4A—C5A	1.373 (11)	C4B—C5B	1.370 (11)
C5A—C6A	1.381 (11)	C5B—C6B	1.384 (11)
C6A—H6A	0.9500	C6B—H6B	0.9500
C7A—C8A	1.500 (12)	C7B—C8B	1.509 (12)
C7A—H7A1	0.9900	C7B—H7B1	0.9900
C7A—H7A2	0.9900	C7B—H7B2	0.9900
C1'A—C6'A	1.399 (10)	C1'B—C2'B	1.373 (12)
C1'A—C2'A	1.400 (12)	C1'B—C6'B	1.393 (10)
C2'A—C3'A	1.380 (12)	C2'B—C3'B	1.391 (12)
C2'A—H2'A	0.9500	C2'B—H2'B	0.9500
C3'A—C4'A	1.389 (11)	C3'B—C4'B	1.364 (10)
C3'A—H3'A	0.9500	C3'B—H3'B	0.9500
C4'A—C5'A	1.369 (12)	C4'B—C5'B	1.392 (12)
C5'A—C6'A	1.388 (11)	C5'B—C6'B	1.399 (11)
C5'A—H5'A	0.9500	C5'B—H5'B	0.9500
C6'A—H6'A	0.9500	C6'B—H6'B	0.9500
			
O4A—S1A—O3A	121.2 (3)	O3B—S1B—O4B	122.7 (3)
O4A—S1A—O2A	109.9 (3)	O3B—S1B—O2B	105.6 (3)
O3A—S1A—O2A	106.0 (3)	O4B—S1B—O2B	110.3 (3)
O4A—S1A—O1A	106.0 (3)	O3B—S1B—O1B	109.4 (3)
O3A—S1A—O1A	110.5 (3)	O4B—S1B—O1B	105.4 (3)
O2A—S1A—O1A	101.4 (3)	O2B—S1B—O1B	101.4 (3)
C4A—O1A—S1A	119.3 (4)	C4B—O1B—S1B	120.0 (4)
C7A—O2A—S1A	117.1 (5)	C7B—O2B—S1B	117.5 (5)
C2A—C1A—C6A	118.1 (7)	C2B—C1B—C6B	120.1 (7)
C2A—C1A—C1'A	121.5 (7)	C2B—C1B—C1'B	119.9 (6)
C6A—C1A—C1'A	120.3 (7)	C6B—C1B—C1'B	120.0 (7)
C3A—C2A—C1A	121.1 (7)	C1B—C2B—C3B	120.8 (7)
C3A—C2A—H2A	119.5	C1B—C2B—H2B	119.6
C1A—C2A—H2A	119.5	C3B—C2B—H2B	119.6
C4A—C3A—C2A	120.0 (8)	C2B—C3B—C4B	119.3 (8)
C4A—C3A—Cl1A	120.1 (6)	C2B—C3B—Cl1B	119.9 (6)
C2A—C3A—Cl1A	119.8 (6)	C4B—C3B—Cl1B	120.7 (6)
C5A—C4A—C3A	119.0 (7)	C5B—C4B—O1B	120.6 (6)
C5A—C4A—O1A	120.1 (6)	C5B—C4B—C3B	119.5 (7)
C3A—C4A—O1A	120.5 (7)	O1B—C4B—C3B	119.8 (7)
C4A—C5A—C6A	121.5 (7)	C4B—C5B—C6B	122.2 (7)
C4A—C5A—Cl2A	118.8 (6)	C4B—C5B—Cl2B	118.7 (6)
C6A—C5A—Cl2A	119.7 (6)	C6B—C5B—Cl2B	119.0 (6)
C5A—C6A—C1A	120.3 (8)	C5B—C6B—C1B	118.1 (7)
C5A—C6A—H6A	119.9	C5B—C6B—H6B	121.0
C1A—C6A—H6A	119.9	C1B—C6B—H6B	121.0
O2A—C7A—C8A	105.4 (6)	O2B—C7B—C8B	108.2 (7)
O2A—C7A—H7A1	110.7	O2B—C7B—H7B1	110.0
C8A—C7A—H7A1	110.7	C8B—C7B—H7B1	110.0
O2A—C7A—H7A2	110.7	O2B—C7B—H7B2	110.0
C8A—C7A—H7A2	110.7	C8B—C7B—H7B2	110.0
H7A1—C7A—H7A2	108.8	H7B1—C7B—H7B2	108.4
C7A—C8A—Cl5A	112.5 (6)	C7B—C8B—Cl5B	108.6 (6)
C7A—C8A—Cl6A	106.7 (6)	C7B—C8B—Cl6B	108.2 (6)
Cl5A—C8A—Cl6A	109.3 (5)	Cl5B—C8B—Cl6B	110.8 (6)
C7A—C8A—Cl4A	110.8 (7)	C7B—C8B—Cl4B	109.5 (7)
Cl5A—C8A—Cl4A	108.6 (5)	Cl5B—C8B—Cl4B	110.0 (5)
Cl6A—C8A—Cl4A	108.8 (5)	Cl6B—C8B—Cl4B	109.7 (5)
C6'A—C1'A—C2'A	118.2 (7)	C2'B—C1'B—C6'B	118.9 (7)
C6'A—C1'A—C1A	120.1 (7)	C2'B—C1'B—C1B	120.7 (6)
C2'A—C1'A—C1A	121.6 (6)	C6'B—C1'B—C1B	120.4 (7)
C3'A—C2'A—C1'A	121.3 (7)	C1'B—C2'B—C3'B	121.2 (7)
C3'A—C2'A—H2'A	119.3	C1'B—C2'B—H2'B	119.4
C1'A—C2'A—H2'A	119.3	C3'B—C2'B—H2'B	119.4
C2'A—C3'A—C4'A	119.3 (8)	C4'B—C3'B—C2'B	119.4 (8)
C2'A—C3'A—H3'A	120.3	C4'B—C3'B—H3'B	120.3
C4'A—C3'A—H3'A	120.3	C2'B—C3'B—H3'B	120.3
C5'A—C4'A—C3'A	120.3 (7)	C3'B—C4'B—C5'B	121.3 (8)
C5'A—C4'A—Cl3A	120.7 (6)	C3'B—C4'B—Cl3B	120.7 (7)
C3'A—C4'A—Cl3A	118.9 (7)	C5'B—C4'B—Cl3B	118.0 (6)
C4'A—C5'A—C6'A	120.7 (7)	C4'B—C5'B—C6'B	118.5 (7)
C4'A—C5'A—H5'A	119.6	C4'B—C5'B—H5'B	120.8
C6'A—C5'A—H5'A	119.6	C6'B—C5'B—H5'B	120.8
C5'A—C6'A—C1'A	120.0 (8)	C1'B—C6'B—C5'B	120.6 (8)
C5'A—C6'A—H6'A	120.0	C1'B—C6'B—H6'B	119.7
C1'A—C6'A—H6'A	120.0	C5'B—C6'B—H6'B	119.7
			
O4A—S1A—O1A—C4A	138.7 (5)	O3B—S1B—O1B—C4B	−4.9 (6)
O3A—S1A—O1A—C4A	5.6 (6)	O4B—S1B—O1B—C4B	−138.6 (6)
O2A—S1A—O1A—C4A	−106.4 (5)	O2B—S1B—O1B—C4B	106.3 (6)
O4A—S1A—O2A—C7A	45.2 (6)	O3B—S1B—O2B—C7B	−177.9 (5)
O3A—S1A—O2A—C7A	177.8 (5)	O4B—S1B—O2B—C7B	−43.3 (6)
O1A—S1A—O2A—C7A	−66.7 (5)	O1B—S1B—O2B—C7B	68.0 (6)
C6A—C1A—C2A—C3A	−1.2 (11)	C6B—C1B—C2B—C3B	3.1 (11)
C1'A—C1A—C2A—C3A	177.5 (7)	C1'B—C1B—C2B—C3B	−178.2 (7)
C1A—C2A—C3A—C4A	0.9 (11)	C1B—C2B—C3B—C4B	−2.3 (11)
C1A—C2A—C3A—Cl1A	−178.0 (6)	C1B—C2B—C3B—Cl1B	179.7 (6)
C2A—C3A—C4A—C5A	1.0 (11)	S1B—O1B—C4B—C5B	91.6 (8)
Cl1A—C3A—C4A—C5A	179.9 (5)	S1B—O1B—C4B—C3B	−92.2 (7)
C2A—C3A—C4A—O1A	174.4 (6)	C2B—C3B—C4B—C5B	1.6 (11)
Cl1A—C3A—C4A—O1A	−6.7 (10)	Cl1B—C3B—C4B—C5B	179.5 (6)
S1A—O1A—C4A—C5A	−92.0 (7)	C2B—C3B—C4B—O1B	−174.6 (6)
S1A—O1A—C4A—C3A	94.7 (7)	Cl1B—C3B—C4B—O1B	3.3 (10)
C3A—C4A—C5A—C6A	−2.6 (11)	O1B—C4B—C5B—C6B	174.4 (6)
O1A—C4A—C5A—C6A	−176.0 (6)	C3B—C4B—C5B—C6B	−1.8 (11)
C3A—C4A—C5A—Cl2A	177.1 (5)	O1B—C4B—C5B—Cl2B	−2.3 (10)
O1A—C4A—C5A—Cl2A	3.7 (9)	C3B—C4B—C5B—Cl2B	−178.5 (5)
C4A—C5A—C6A—C1A	2.3 (11)	C4B—C5B—C6B—C1B	2.5 (11)
Cl2A—C5A—C6A—C1A	−177.4 (5)	Cl2B—C5B—C6B—C1B	179.2 (5)
C2A—C1A—C6A—C5A	−0.4 (11)	C2B—C1B—C6B—C5B	−3.2 (11)
C1'A—C1A—C6A—C5A	−179.1 (6)	C1'B—C1B—C6B—C5B	178.2 (6)
S1A—O2A—C7A—C8A	−148.3 (6)	S1B—O2B—C7B—C8B	148.2 (6)
O2A—C7A—C8A—Cl5A	58.7 (8)	O2B—C7B—C8B—Cl5B	−180.0 (5)
O2A—C7A—C8A—Cl6A	178.5 (5)	O2B—C7B—C8B—Cl6B	−59.6 (8)
O2A—C7A—C8A—Cl4A	−63.2 (7)	O2B—C7B—C8B—Cl4B	59.9 (8)
C2A—C1A—C1'A—C6'A	36.2 (11)	C2B—C1B—C1'B—C2'B	145.8 (8)
C6A—C1A—C1'A—C6'A	−145.1 (7)	C6B—C1B—C1'B—C2'B	−35.5 (11)
C2A—C1A—C1'A—C2'A	−140.7 (8)	C2B—C1B—C1'B—C6'B	−34.4 (11)
C6A—C1A—C1'A—C2'A	38.0 (11)	C6B—C1B—C1'B—C6'B	144.2 (7)
C6'A—C1'A—C2'A—C3'A	0.1 (12)	C6'B—C1'B—C2'B—C3'B	0.8 (12)
C1A—C1'A—C2'A—C3'A	177.0 (7)	C1B—C1'B—C2'B—C3'B	−179.4 (7)
C1'A—C2'A—C3'A—C4'A	−0.5 (12)	C1'B—C2'B—C3'B—C4'B	0.0 (13)
C2'A—C3'A—C4'A—C5'A	1.7 (12)	C2'B—C3'B—C4'B—C5'B	−0.5 (12)
C2'A—C3'A—C4'A—Cl3A	−177.9 (6)	C2'B—C3'B—C4'B—Cl3B	178.5 (6)
C3'A—C4'A—C5'A—C6'A	−2.4 (12)	C3'B—C4'B—C5'B—C6'B	0.3 (12)
Cl3A—C4'A—C5'A—C6'A	177.1 (6)	Cl3B—C4'B—C5'B—C6'B	−178.8 (6)
C4'A—C5'A—C6'A—C1'A	2.0 (12)	C2'B—C1'B—C6'B—C5'B	−1.1 (12)
C2'A—C1'A—C6'A—C5'A	−0.8 (11)	C1B—C1'B—C6'B—C5'B	179.2 (7)
C1A—C1'A—C6'A—C5'A	−177.8 (7)	C4'B—C5'B—C6'B—C1'B	0.5 (11)
